# Reliability of the gamma index analysis as a verification method of volumetric modulated arc therapy plans

**DOI:** 10.1186/s13014-018-1123-x

**Published:** 2018-09-14

**Authors:** Jong Min Park, Jung-in Kim, So-Yeon Park, Do Hoon Oh, Sang-Tae Kim

**Affiliations:** 1 Department of Radiation Oncology, Seoul National University Hospital, Seoul, South Korea; 2 Institute of Radiation Medicine, Seoul National University Medical Research Center, Seoul, South Korea; 3 Biomedical Research Institute, Seoul National University Hospital, Seoul, South Korea; 4 Institute for Smart System, Robotics Research Laboratory for Extreme Environments, Advanced Institutes of Convergence Technology, Suwon, South Korea; 5 Department of Radiation Oncology, Veterans Health Service Medical Center, Seoul, South Korea; 6 Department of Radiation Oncology, Myongji Hospital, Goyang, South Korea; 7 Nuclear Emergency Division, Radiation Protection and Emergency Preparedness Bureau, Nuclear Safety and Security Commission, Seoul, South Korea

**Keywords:** Gamma passing rate, Volumetric modulated arc therapy, Patient-specific quality assurance, Log file analysis, Modulation degree

## Abstract

**Background:**

We investigate the gamma passing rate (GPR) consistency when applying different types of gamma analyses, linacs, and dosimeters for volumetric modulated arc therapy (VMAT).

**Methods:**

A total of 240 VMAT plans for various treatment sites, which were generated with Trilogy (140 plans) and TrueBeam STx (100 plans), were retrospectively selected. For each VMAT plan, planar dose distributions were measured with both MapCHECK2 and ArcCHECK dosimeters. During the planar dose distribution measurements, the actual multileaf collimator (MLC) positions, gantry angles, and delivered monitor units were recorded and compared to the values in the original VMAT plans to calculate mechanical errors. For each VMAT plan, both the global and local gamma analyses were performed with 3%/3 mm, 2%/2 mm, 2%/1 mm, 1%/2 mm, and 1%/1 mm. The Pearson correlation coefficients (*r*) were calculated 1) between the global and the local GPRs, 2) between GPRs with the MapCHECK2 and the ArcCHECK dosimeters, 3) and between GPRs and the mechanical errors during the VMAT delivery.

**Results:**

For the MapCHECK2 measurements, strong correlations between the global and local GPRs were observed only with 1%/2 mm and 1%/1 mm (*r* > 0.8 with *p* < 0.001), while weak or no correlations were observed for the ArcCHECK measurement. Between the MapCHECK2 and ArcCHECK measurements, the global GPRs showed no correlations (all with *p* > 0.05), while the local GPRs showed moderate correlations only with 2%/1 mm and 1%/1 mm for TrueBeam STx (*r* > 0.5 with *p* < 0.001). Both the global and local GPRs always showed weak or no correlations with the MLC positional errors except for the GPRs of MapCHECK2 with 1%/2 mm and 1%/1 mm for TrueBeam STx and the GPR of ArcCHECK with 1%/2 mm for Trilogy (*r* < − 0.5 with *p* < 0.001).

**Conclusions:**

The GPRs varied according to the types of gamma analyses, dosimeters, and linacs. Therefore, each institution should carefully establish their own gamma analysis protocol by determining the type of gamma index analysis and the gamma criterion with their own linac and their own dosimeter.

## Background

Intensity-modulated radiation therapy (IMRT) enables conformal delivery of prescription doses to target volumes while minimizing radiotherapy-induced complications by reducing the doses to organs at risk (OARs) located nearby the target volumes [1, 2]. This is possible owing to photon beam intensity modulations by mechanical modulations of the multileaf collimator (MLC) positions [3]. Compared to IMRT, volumetric modulated arc therapy (VMAT) is possible to deliver equal to or better dose distributions than those of IMRT more rapidly by simultaneous mechanical modulations of the MLC positions, gantry rotation speeds, and dose rates [4]. The optimal dose distributions of VMAT are also attributed to photon beam modulations. Although the photon beam modulation of VMAT enables to generate an optimal dose distribution, an excessive modulation of a VMAT plan results in discordance between the planned and actually delivered dose distributions to a patient, i.e., patients are not treated as intended with excessively modulated VMAT plans [5–7]. This is because of increases in the dose calculation uncertainty and mechanical uncertainty [5, 6, 8]. For excessively modulated VMAT plans, small or irregularly shaped beam apertures tend to be used more frequently, whose dose calculation accuracy with current dose calculation algorithms is limited; therefore, the calculated dose distribution (intended dose distribution) would not be actually delivered to a patient during treatment due to the increased dose calculation uncertainty [5, 6]. Besides the dose calculation uncertainty of excessively modulated VMAT plans, mechanical movements of MLCs, gantry rotation, and beam delivery system become complicated, which increases the mechanical uncertainty [8–10]. Therefore, the intended mechanical movements of excessively modulated VMAT plans might not be perfectly implemented during the actual plan delivery to a patient. This also causes discordance between the planned and delivered dose distributions. In this respect, pre-treatment verification of the delivered dose distributions of VMAT plans, i.e., pre-treatment patient-specific quality assurance (QA), is highly recommended and routinely performed for each VMAT plan before treatment in the clinical setting [11–13]. The most popular method as patient-specific QA for VMAT is the gamma index analysis [14–16].

Although the gamma index analysis is widely adopted in the clinical setting, some issues on the gamma index analysis have been raised by previous studies [11, 13, 17–19]. The gamma index method evaluates the coincidence between the calculated and measured dose distributions by utilizing the percent dose difference (DD) and distance to agreement [15]. Regarding the calculation of the DD, there are two types of gamma index methods, which are the global and local gamma index analyses [1]. The global gamma index analysis calculates the DDs relative to the maximum dose (or prescription dose), while the local gamma index analysis calculates the DDs relative to the doses at each evaluated point. Because the DD is a percent value, the local gamma index analysis could exaggerate the DDs in the low-dose regions, while the global gamma index method could underestimate the dose discrepancies in the low-dose regions [1]. Since there are no clear guidelines to use the global or local gamma index analysis as pre-treatment patient-specific QA for VMAT, each clinic autonomously decides that which type of gamma index analysis should be used in that clinic; however, it is uncertain that the global gamma index analysis can substitute for the local gamma index analysis or vice versa. For the tolerance levels of the gamma passing rates, Heilemann et al. demonstrated that a global gamma passing rate of 90% with 2%/2 mm could detect clinically unacceptable VMAT plans owing to significant errors by introducing deliberately MLC errors [12]. However, it was also demonstrated that the gamma passing rates of IMRT or VMAT depend on the types of dosimeter [11, 13]. Hussein et al. and Fredh et al. showed dependency of the gamma passing rates on the types of dosimeters for IMRT and VMAT plans [11, 13]. They claimed that the configuration and resolution of the detector have a great impact on the calculation of the gamma passing rates. Therefore, the tolerance levels suggested by previous studies or international guidelines might not be always appropriate for a particular institution’s dosimeter. Moreover, it is uncertain that the gamma index analysis could sensitively reflect the VMAT delivery accuracy depending on the modulation degree. Although the gamma index analysis is widely accepted in the clinical setting, its reliability seems to be investigated thoroughly. Therefore, in this study, we investigated the correlation between the global and local gamma passing rates by utilizing 240 VMAT plans with various modulation degrees. In addition, by utilizing two types of dosimeters, we investigated the correlation between the gamma passing rates of those two dosimeters. We also investigated the relationship between the gamma passing rates and the mechanical errors acquired from the log files recorded during the VMAT delivery in the linac control system. Finally, we investigated the relevance between the gamma passing rates and the differences in the dose-volumetric parameters between the original plans and the plans reconstructed with the log files. By doing this, we evaluated the reliability of the gamma index analysis under various conditions.

## Methods

### Patient selection

For this study, a total of 200 patients with head and neck (H&N) cancer (60 patients), prostate cancer (40 patients), liver cancer (11 patients treated with fractionated radiotherapy and 20 patients treated with stereotactic ablative radiotherapy (SABR)), lung cancer (20 patients treated with SABR), brain tumor (20 patients), and spine tumor (9 patients treated with fractionated radiotherapy and 20 patients with SABR) were retrospectively selected after an institutional review board approval (IRB No. 1802–069-922). Every patient underwent CT scans using Brilliance CT Big Bore™ (Philips, Amsterdam, the Netherlands).

### Generation of VMAT plans

A single VMAT plan was generated for each patient except for the patients with prostate cancer. For those patients, two VMAT plans per a patient were generated, which were a primary plan and a boost plan. Plan information is summarized in Table 1. A total of 140 VMAT plans were generated using the C-series linac, Trilogy™ with the Millennium 120™ MLC (Varian Medical Systems, Palo Alto, CA) while a total of 100 VMAT plans were generated with TrueBeam STx™ with the high-definition (HD) 120™ MLC (Varian Medical Systems, Palo Alto, CA, USA). Every VMAT plan in this study was generated with the Eclipse™ system (Varian Medical Systems, Palo Alto, CA). A progressive resolution optimizer (PRO, ver.13.7, Varian Medical Systems, Palo Alto, CA) was used for optimization of the VMAT plans, and the anisotropic analytic algorithm (AAA, ver.13.7, Varian Medical Systems, Palo Alto) was used for dose calculation with a dose calculation resolution of 1 mm.

**Table 1:** - Plan information

	*N*	Energy	Prescription dose	Fraction number
Trilogy (*N* = 140)				
H&N	40	6 MV	67.5 Gy to PTV_67.5Gy_ 54 Gy to PTV_54Gy_ 48 Gy to PTV_48Gy_	30
Prostate primary	40	15 MV	50.4	28
Prostate boost	40	15 MV	30.6	17
Liver	11	15 MV	50	25
Spine	9	15 MV	30	10
TrueBeam STx (*N* = 100)				
H&N	20	6 MV	67.5 Gy to PTV_67.5Gy_ 54 Gy to PTV_54Gy_ 48 Gy to PTV_48Gy_	30
Brain	20	6 MV	30	10
Lung SABR	20	6 MV FFF	60	4
Spine SABR	20	10 MV FFF	19	1
Liver SABR	20	10 MV FFF	42	3

Note: *H&N* Head and neck, *SABR* Stereotactic ablative radiotherapy, *FFF* Flattening filter free

### Gamma index analysis

For the gamma index analysis of each VMAT plan, two types of dosimeters were utilized in this study, which were a MapCHECK2™ dosimeter inserted in a MapPHAN™ (Sun Nuclear Corporation, Melbourne, FL) and an ArcCHECK™ dosimeter (Sun Nuclear Corporation, Melbourne, FL). To generate the reference 2D dose distribution, CT images of both dosimeters were taken and used to generate verification plans in the Eclipse system. The dose distributions were calculated with a dose calculation resolution of 1 mm, which is the finest dose calculation resolution of the Eclipse system. To reduce the volume averaging effect, we calculated the dose distributions with the finest dose calculation resolution [20]. To acquire accurate measured dose distributions, both the MapCHECK2 and ArcCHECK dosimeters were calibrated according to the manufacturer guidelines. Before measuring the 2D dose distributions, the outputs of Trilogy and TrueBeam STx were calibrated according to the American Association of Physicists in Medicine Task Group 51 protocol [21]. For an accurate setup of the dosimeters, cone beam computed tomography images of the dosimeters were acquired to verify the dosimeter setup before the measurements. Both the global and local gamma passing rates with various gamma criteria, which were 3%/3 mm, 2%/2 mm, 2%/1 mm, 1%/2 mm, and 1%/1 mm, were calculated with the absolute doses using the SNC patient™ software (Sun Nuclear Corporation, Melbourne, FL), which linearly interpolates calculated doses less than 1 mm resolution [22]. The SNC patient software was used to calculate gamma passing rates both with the MapCHECK2 measurements and the ArcCHECK measurements. When calculating gamma passing rates, the reference images were measured dose distributions and the evaluated images were calculated dose distributions. To calculate the global gamma passing rates, we calculated the DDs relative to the maximum doses.

### Differences in the mechanical parameters between the original VMAT plans and the log files recorded during the VMAT delivery

When measuring the 2D dose distributions of the VMAT plans with the MapCHECK2 and ArcCHECK dosimeters, the log files in the linac control system, which are the records of the actual MLC positions, gantry angles, and delivered monitor units (MUs) at each control point during the VMAT delivery, were acquired. The reliability of the log files was examined by periodic linac QA by international guidelines and previous studies [23–25]. The MU delivery errors at each control point were considered mechanical errors in this study since the MU delivery at each control point is controlled by the linac control system synchronized with the MLC movements and gantry rotation speeds. To compare the values of the mechanical parameters from the log files to the values defined in the original VMAT plans, the log files were reformatted as DICOM-RT files with an in-house program written in MATLAB (Mathworks Inc., Natick, MA). The differences in the MLC positions, gantry angles, and delivered MUs were calculated with the DICOM-RT formatted log files and the original VMAT plans in the DICOM-RT format. Because two log file sets were acquired during the VMAT deliveries for the MapCHECK2 and ArcCHECK measurements, the two sets of the calculated differences were averaged.

### Modulation complexity score and the differences in the dose-volumetric parameters between the original VMAT plans and the reconstructed VMAT plans with the log files

We calculated modulation complexity score for VMAT (MCS_v_) suggested by Masi et al. to quantify the modulation degrees of each VMAT plan [9]. The MCS_v_ has values in the range from 0 to 1. As increasing the modulation complexity of a VMAT plan, the value of MCS_v_ decreases (cf. MCS_v_ = 1 means no modulation of a VMAT plan).

The DICOM-RT formatted files were imported to the Eclipse system and dose distributions were calculated with the patient CT images. After that, we calculated the minimum dose to cover 95% of the planning target volume (PTV) (D_95%_) as well as the percent volume of the body structure irradiated by at least 50% of the prescription dose (V_50%_). Since the H&N VMAT plan had a total of three PTVs, only the PTV receiving the highest prescription dose (PTV_67.5Gy_) was analyzed. The values of D_95%_ of the PTV and V_50%_ of the body calculated from the reconstructed plans with the log files were compared to those of the original VMAT plans.

### Correlation analysis

To evaluate the consistency between the global and the local gamma passing rates, the Pearson correlation coefficient (*r*) with corresponding *p* values was calculated between the global and local gamma passing rates with various gamma criteria. The correlations with *r* values from 0.3 to 0.49 are regarded as weak correlations. Those from 0.5 to 0.69 and 0.7 to 1.0 are regarded as moderate and strong correlations, respectively. The *p* values equal to or less than 0.05 are regarded as statistically significant in this study. To evaluate the dosimeter dependency of the gamma passing rates, the correlations between the gamma passing rates from the MapCHECK2 measurements and those from the ArcCHECK measurements were analyzed. To evaluate the detectability of the gamma passing rates on the mechanical errors during the VMAT deliveries, the correlations between the gamma passing rates and the mechanical errors calculated with the original VMAT plans and the log files were analyzed. The correlations between the gamma passing rates and the MCS_v_ were calculated to examine changes in the gamma passing rates according to the modulation degree of VMAT plans [9]. To examine relevance of the gamma passing rates to the changes in the dose-volumetric parameters, the correlations between the gamma passing rates and the differences in the PTV D_95%_ of the original treatment plans from those of the reconstructed plans with the log files were analyzed. In addition, the correlations between the gamma passing rates and the differences in the V_50%_ of the body structures of the original treatment plans from those of the reconstructed plans with log files were also analyzed.

## Results

### Global gamma passing rates

**Fig. 1:**
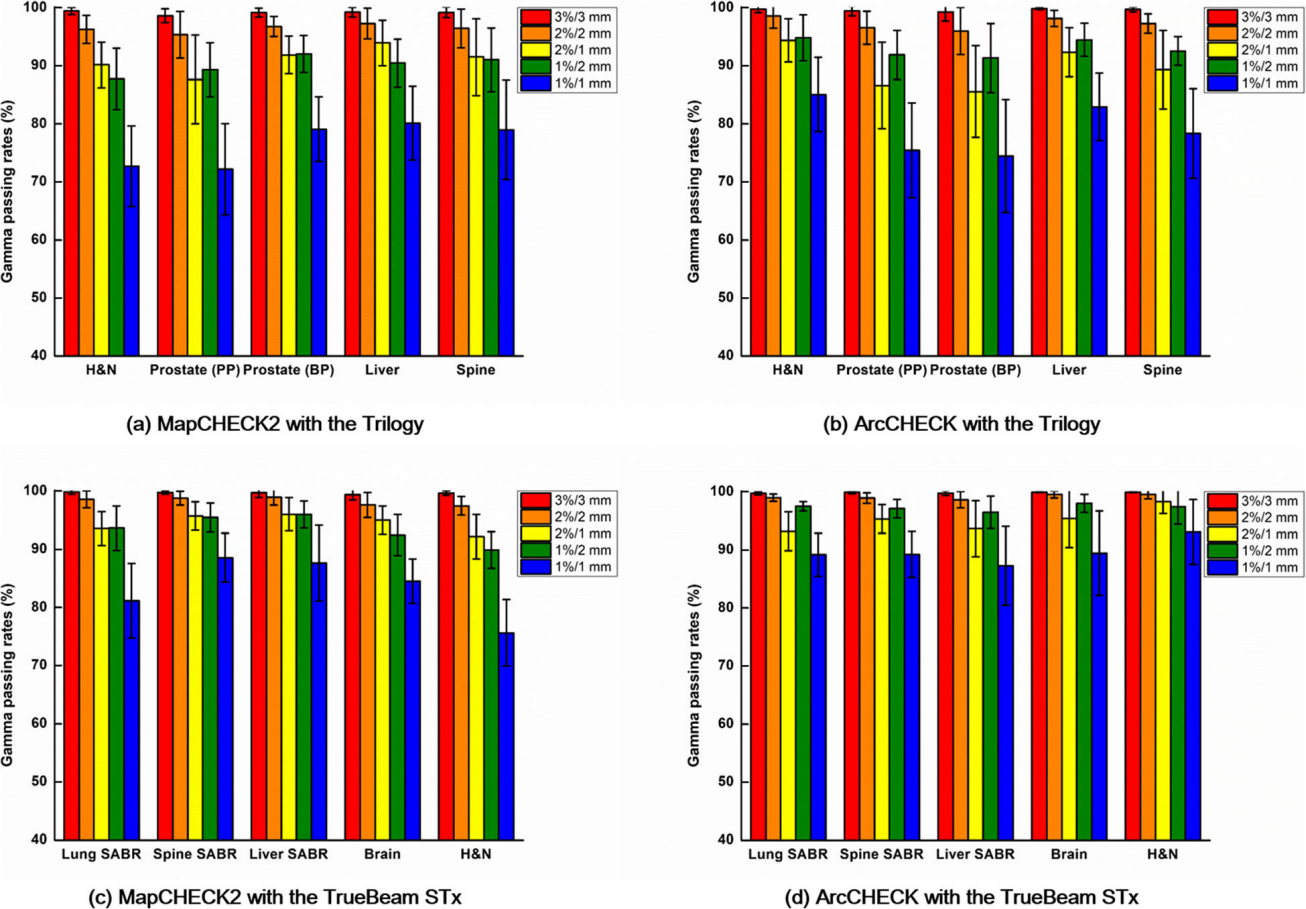
Global gamma passing rates of the VMAT plans. Global gamma passing rates with various gamma criteria calculated from the measurements with the MapCHECK2 dosimeter are shown for Trilogy (**a**) and TrueBeam STx (**c**). Those with the ArcCHECK dosimeter using Trilogy (**b**) and TrueBeam STx (**d**) are also shown. For the global gamma passing rates with Trilogy, the H&N plans, prostate primary plans (PPs), prostate boost plans (BPs), liver plans, and spine plans were analyzed, while the lung SABR, spine SABR, liver SABR, brain, and H&N plans were analyzed with TrueBeam STx

The global gamma passing rates with various gamma criteria of the VMAT plans for various treatment sites, measured with the MapCHECK2 and ArcCHECK dosimeters, are shown in Fig. 1.

For the VMAT plans with Trilogy, the prostate primary plans showed the lowest gamma passing rates in general with the MapCHECK2 measurements. For the ArcCHECK measurements, the prostate boost plans showed the lowest gamma passing rates with every gamma criterion tested in this study. The gamma passing rates with MapCHECK2 were not coincident with those with ArcCHECK, sometimes contradictory to each other.

For the VMAT plans with TrueBeam STx, the H&N plans generally showed the lowest gamma passing rates with the MapCHECK2 measurements, while the liver plans generally showed the highest gamma passing rates. For the ArcCHECK measurements, the lung SABR plans generally showed the lowest gamma passing rates, while the H&N plans generally showed the highest gamma passing rates. The gamma passing rates with MapCHECK2 were not coincident with those with ArcCHECK, sometimes contradictory to each other.

### Local gamma passing rates

**Fig. 2:**
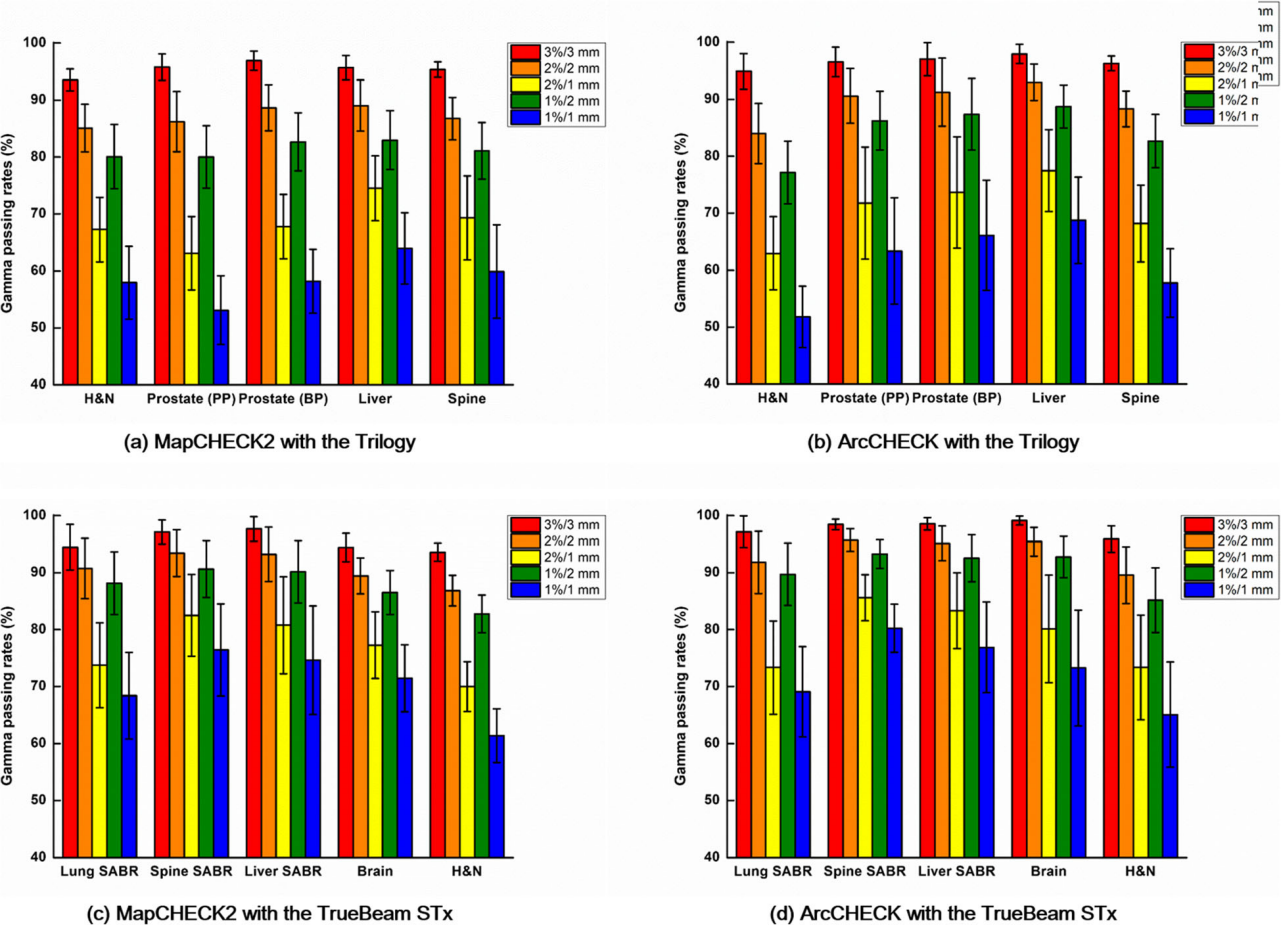
Local gamma passing rates of the VMAT plans. Local gamma passing rates with various gamma criteria calculated from the measurements with the MapCHECK2 dosimeter are shown for Trilogy (**a**) and TrueBeam STx (**c**). Those with the ArcCHECK dosimeter using Trilogy (**b**) and TrueBeam STx (**d**) are also shown. For the local gamma passing rates with Trilogy, H&N plans, prostate primary plans (PPs), prostate boost plans (BPs), liver plans, and spine plans were analyzed, while the lung SABR, spine SABR, liver SABR, brain, and H&N plans were analyzed with TrueBeam STx

The local gamma passing rates with various gamma criteria of the VMAT plans for various treatment sites, measured with MapCHECK2 and ArcCHECK, are shown in Fig. 2.

For the VMAT plans with Trilogy, the H&N plans showed the lowest gamma passing rates with 3%/3 mm and 2%/2 mm, while the prostate primary plans showed the lowest gamma passing rates with the rest of the gamma criteria for the MapCHECK2 measurements. However, for the ArcCHECK measurements, the H&N VMAT plans showed the lowest gamma passing rates consistently regardless of the gamma criteria. Both the MapCHECK2 and the ArcCHECK measurements showed the highest gamma passing rates for the liver plans in general. The gamma passing rates with MapCHECK2 were generally coincident with those with those of ArcCHECK, however, not always.

For the VMAT plans with TrueBeam STx, both the MapCHECK2 and ArcCHECK measurements showed the lowest gamma passing rates for the H&N plans. Except for the gamma passing rates with 3%/3 mm, both the MapCHECK2 and ArcCHECK measurements showed the highest gamma passing rates for the spine SABR VMAT plans. Similar to the results with Trilogy, the gamma passing rates of MapCHECK2 were generally coincident with those of ArcCHECK.

### Mechanical errors during the VMAT delivery

**Fig. 3:**
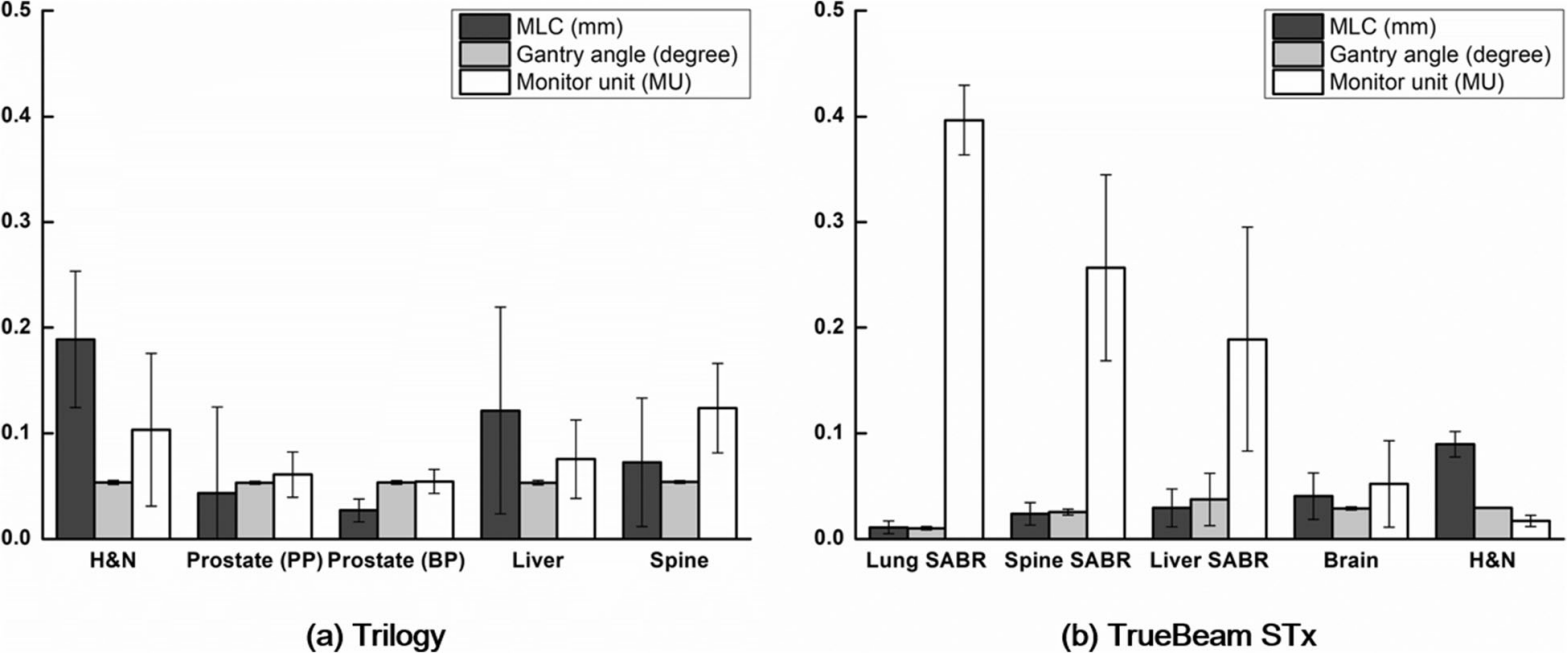
Differences in the mechanical parameters between those defined in the original VMAT plans and those recorded during the VMAT plan deliveries in the log files. MLC positional errors, gantry angle errors, and MU errors are shown for Trilogy (**a**) and TrueBeam STx (**b**). The units of the MLC errors, gantry angle errors, and MU errors are millimeters, degrees, and MUs, respectively

The differences in the mechanical parameters between the values defined in the original VMAT plans and those in the log files recorded during the VMAT deliveries are shown in Fig. 3.

For the mechanical errors of Trilogy, the MLC positioning errors were the largest when delivering the H&N VMAT plans (0.19 ± 0.06 mm), while those differences were the lowest when delivering the prostate boost plans (0.03 ± 0.01 mm). The MU delivery errors were the largest for the spine VMAT plans (0.12 ± 0.04 MU), while those were the smallest for the prostate boost plans (0.05 ± 0.01 mm).

For the mechanical errors of TrueBeam STx, the MLC positioning errors were the largest for the H&N VMAT plans (0.09 ± 0.01 mm) same as the results with Trilogy, while those errors were the smallest for the lung SABR VMAT plans (0.01 ± 0.01 mm). However, this was opposite for the MU delivery errors showing the largest errors during the lung SABR VMAT deliveries (0.40 ± 0.03 MU), while showing the smallest errors during the H&N VMAT deliveries (0.02 ± 0.01 MU). The large MU delivery errors were only observed in the SABR VMAT plans. The gantry positional error was the largest for the liver SABR VMAT plans (0.04 ± 0.02°), while that was the smallest for the lung SABR VMAT plans (0.01 ± 0.00°).

In general, the MLC positional errors and the gantry positional errors of TrueBeam STx were much smaller than those of Trilogy. The MU delivery errors of the SABR VMAT plans with TrueBeam STx were generally larger than those with the non-SABR VMAT plans both with TrueBeam STx and Trilogy.

### Modulation complexity score for VMAT

**Fig. 4:**
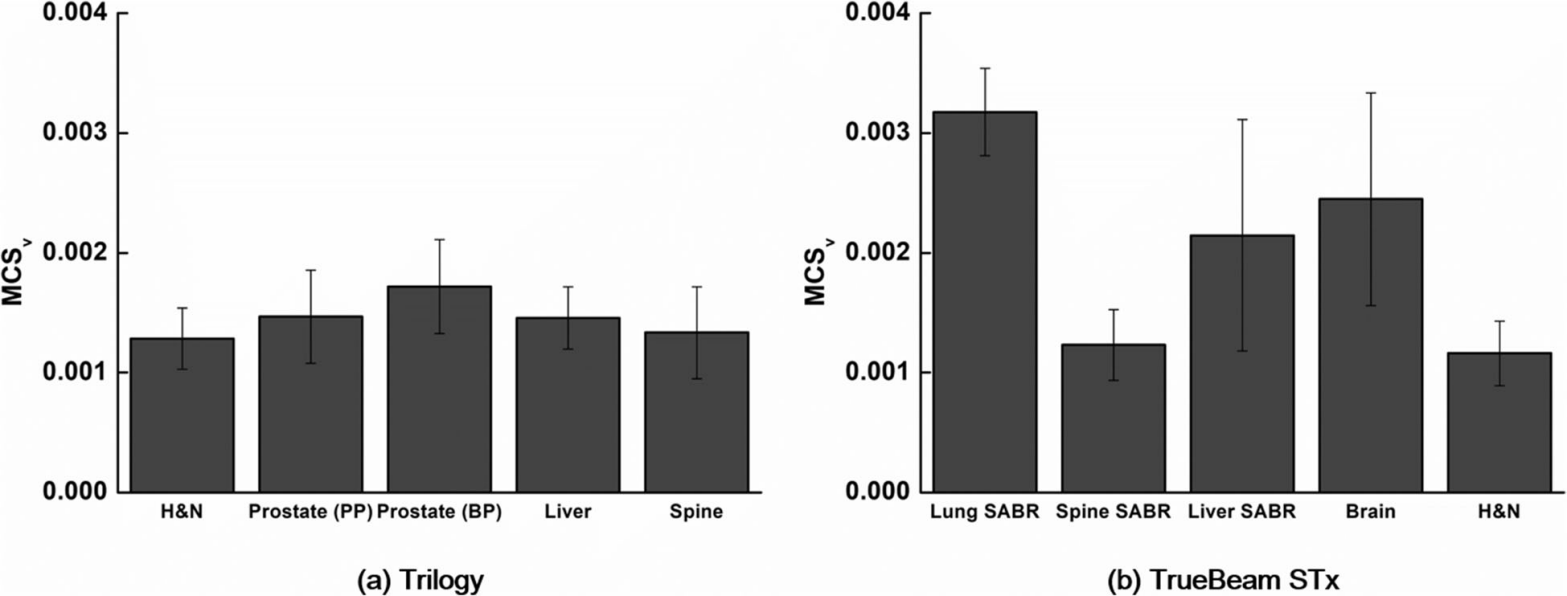
Modulation complexity score for VMAT plans. Average values of the modulation complexity score for volumetric modulated arc therapy (MCS_v_) for each treatment site are shown. The MCS_v_ of plans with the Trilogy (**a**) and the TrueBeam STx (**b**) are shown

The average values of MCS_v_ calculated from VMAT plans with the Trilogy and TrueBeam STx are shown in Fig. 4. For the Trilogy, the H&N VMAT plans showed the lowest average value of MCS_v_ (1.28 × 10^− 3^ ± 0.26 × 10^− 3^) while the prostate boost VMAT plans showed the highest average value of MCS_v_ (1.72 × 10^− 3^ ± 0.39 × 10^− 3^). For the TrueBeam STx, the H&N VMAT plans showed the lowest average value of MCS_v_ (1.16 × 10^− 3^ ± 0.27 × 10^− 3^) while the lung SABR VMAT plans showed the highest average value of MCS_v_ (3.17 × 10^− 3^ ± 0.36 × 10^− 3^).

### Correlations between the global and local gamma passing rates

The Pearson correlation coefficients (*r*) between the global and local gamma passing rates are shown in Table 2 with corresponding *p* values.

**Table 2:** - Correlations between the global and local gamma passing rates

Gamma criteria	MapCHECK2 measurements		ArcCHECK measurements	
	*r*	*p*	*r*	*p*
Trilogy (C-series linac)				
3%/3 mm	0.218	0.010	0.540	< 0.001
2%/2 mm	0.621	< 0.001	0.440	< 0.001
2%/1 mm	0.642	< 0.001	0.339	< 0.001
1%/2 mm	0.806	< 0.001	0.469	< 0.001
1%/1 mm	0.770	< 0.001	0.325	< 0.001
TrueBeam STx (SABR + non-SABR)				
3%/3 mm	–	–	0.271	0.006
2%/2 mm	0.451	< 0.001	0.202	0.043
2%/1 mm	0.510	< 0.001	0.376	< 0.001
1%/2 mm	0.860	< 0.001	0.391	< 0.001
1%/1 mm	0.842	< 0.001	0.383	< 0.001
TrueBeam STx (SABR)				
3%/3 mm	–	–	0.409	0.001
2%/2 mm	0.332	0.010	0.318	0.013
2%/1 mm	0.531	< 0.001	0.519	< 0.001
1%/2 mm	0.845	< 0.001	0.460	< 0.001
1%/1 mm	0.851	< 0.001	0.480	< 0.001
TrueBeam STx (non-SABR)				
3%/3 mm	–	–	–	–
2%/2 mm	0.434	0.005	–	–
2%/1 mm	0.404	0.010	0.454	0.003
1%/2 mm	0.826	< 0.001	0.453	0.003
1%/1 mm	0.758	< 0.001	0.506	0.001

Note: Only *r* values with *p* ≤ 0.05 are shown

For the MapCHECK2 measurements, the strongest correlations were observed between the global and local gamma passing rates with a gamma criterion of 1%/2 mm for both the Trilogy and TrueBeam STx systems (*r* = 0.806 with *p* < 0.001 for Trilogy and *r* = 0.860 with *p* < 0.001 for TrueBeam STx). The tighter the gamma criteria, the stronger the correlations became in general. With gamma criteria of 2%/1 mm, 1%/2 mm, and 1%/1 mm, moderate or strong correlations were observed between the global and local gamma passing rates with both the Trilogy and TrueBeam STx systems, showing *r* values larger than 0.5 (all with *p* < 0.001). No correlations were observed between the global and local gamma passing rates with 3%/3 mm. For the TrueBeam STx, no distinctive differences in the correlations were observed between SABR and non-SABR VMAT plans.

For the ArcCHECK measurements, weak or no correlations between the global and local gamma passing rates were observed for every gamma criterion except for 3%/3 mm. With a gamma criterion of 3%/3 mm with the Trilogy system, a moderate correlation (*r* = 0.540 with *p* < 0.001) was observed between the global and local gamma passing rates. For the SABR plans of the TrueBeam STx, a moderate correlation (*r* = 0.519 with *p* < 0.001) was observed between the global and local gamma passing rates with 2%/1 mm. No clear tendency was observed in the correlations between the global and local gamma passing rates with both the Trilogy and TrueBeam STx systems for the ArcCHECK measurements.

### Correlations between the gamma passing rates of the MapCHECK2 and ArcCHECK measurements

The *r* values between the gamma passing rates of the MapCHECK2 and ArcCHECK measurements are shown in Table 3 with corresponding *p* values.

**Table 3:** - Correlations between the gamma passing rates of MapCHECK2 and ArcCHECK

Gamma criteria	Global gamma passing rates		Local gamma passing rates	
	*r*	*p*	*r*	*p*
Trilogy (C-series linac)				
3%/3 mm	–	–	0.242	0.004
2%/2 mm	–	–	0.288	0.001
2%/1 mm	–	–	0.260	0.002
1%/2 mm	–	–	0.266	0.001
1%/1 mm	–	–	0.202	0.016
TrueBeam STx (SABR + non-SABR)				
3%/3 mm	–	–	0.365	< 0.001
2%/2 mm	–	–	0.412	< 0.001
2%/1 mm	–	–	0.528	< 0.001
1%/2 mm	–	–	0.468	< 0.001
1%/1 mm	–	–	0.589	< 0.001
TrueBeam STx (SABR)				
3%/3 mm	–	–	0.355	0.005
2%/2 mm	–	–	0.263	0.042
2%/1 mm	0.273	0.035	0.381	0.003
1%/2 mm	–	–	0.275	0.033
1%/1 mm	–	–	0.413	0.001
TrueBeam STx (non-SABR)				
3%/3 mm	–	–	0.369	0.019
2%/2 mm	–	–	0.629	< 0.001
2%/1 mm	–	–	0.729	< 0.001
1%/2 mm	–	–	0.637	< 0.001
1%/1 mm	–	–	0.745	< 0.001

Note: Only *r* values with *p* ≤ 0.05 are shown

For the global gamma passing rates, no correlations between the gamma passing rates of the MapCHECK2 and ArcCHECK measurements were observed for every gamma criterion (all with *r* < 0.3).

For the local gamma passing rates with the TrueBeam STx system, moderate correlations were observed between the gamma passing rates of the MapCHECK2 and ArcCHECK measurements with 2%/1 mm and 1%/1 mm (*r* = 0.528 with *p* < 0.001 for 2%/1 mm and *r* = 0.589 with *p* < 0.001 for 1%/1 mm). For the rest of the gamma passing rates with TrueBeam STx, weak correlations were observed (3%/3 mm, 2%/2 mm, and 1%/2 mm). To examine correlations of the non-SABR plans separately, strong correlations were observed except for the gamma passing rates with 3%/3 mm (all with *r* > 0.6 with *p* < 0.001). For the Trilogy system, no correlations were observed between the gamma passing rates of the two dosimeters.

### Correlations between the gamma passing rates and the mechanical errors

The *r* values between the gamma passing rates and the MLC positional errors during the VMAT delivery are shown in Table 4 with corresponding *p* values.

**Table 4:** - Correlations between the gamma passing rates and the MLC positional errors

Gamma criteria	MapCHECK2 measurements				ArcCHECK measurements			
	Global gamma passing rates		Local gamma passing rates		Global gamma passing rates		Local gamma passing rates	
	*r*	*p*	*r*	*p*	*r*	*p*	*r*	*p*
Trilogy (C-series linac)								
3%/3 mm	0.213	0.011	− 0.447	< 0.001	–	–	–	–
2%/2 mm	–	–	− 0.183	0.031	0.307	< 0.001	−0.355	< 0.001
2%/1 mm	–	–	0.190	0.025	0.536	< 0.001	− 0.211	0.013
1%/2 mm	− 0.334	< 0.001	–	–	0.305	< 0.001	− 0.503	< 0.001
1%/1 mm	–	–	–	–	0.546	< 0.001	−0.374	< 0.001
TrueBeam STx (SABR + non-SABR)								
3%/3 mm	–	–	−0.339	0.001	–	–	−0.382	< 0.001
2%/2 mm	−0.358	< 0.001	−0.478	< 0.001	0.211	0.035	−0.369	< 0.001
2%/1 mm	−0.328	0.001	−0.394	< 0.001	0.458	< 0.001	−0.230	0.021
1%/2 mm	−0.554	< 0.001	−0.542	< 0.001	–	–	−0.478	< 0.001
1%/1 mm	−0.531	< 0.001	−0.502	< 0.001	0.316	0.001	−0.364	< 0.001
TrueBeam STx (non-SABR)								
3%/3 mm	–	–	–	–	–	–	−0.699	< 0.001
2%/2 mm	−0.336	0.034	−0.475	0.002	–	–	−0.593	< 0.001
2%/1 mm	−0.507	0.001	−0.470	0.002	0.463	0.003	–	–
1%/2 mm	−0.577	< 0.001	−0.584	< 0.001	–	–	− 0.619	< 0.001
1%/1 mm	− 0.740	< 0.001	− 0.622	< 0.001	0.348	0.028	−0.365	0.021

Note: Only *r* values with *p* ≤ 0.05 are shown

For the MapCHECK2 measurements with Trilogy, both the global and local gamma passing rates always showed weak or no correlations with the MLC positional errors. In the case of TrueBeam STx, both the global and local gamma passing rates also showed weak or no correlations with the MLC positional errors except for the gamma passing rates with 1%/2 mm and 1%/1 mm, which showed moderate correlations (*r* < − 0.5 with *p* < 0.001). To examine correlations of the non-SABR plans separately, a strong correlation was observed for the global gamma passing rates with 1%/1 mm (*r* = − 0.740 with *p* < 0.001). The correlations of the non-SABR plans were generally higher than those of the SABR plans.

For the ArcCHECK measurements with Trilogy, the global gamma passing rates with 2%/1 mm and 1%/1 mm showed moderate correlations with the MLC errors (*r* = 0.536 with *p* < 0.001 for 2%/1 mm and *r* = 0.546 with *p* < 0.001 for 1%/1 mm). However, this is not reasonable because the *r* values were positive. Since the gamma passing rates should decrease with increasing the MLC errors, there should be negative correlations between the gamma passing rates and the MLC errors. The local gamma passing rate with 1%/2 mm showed a moderate negative correlation with the MLC errors (*r* = − 0.503 with *p* < 0.001). In the case of TrueBeam STx, weak or no correlations were observed between the gamma passing rates and the MLC errors. To examine non-SABR plans separately, correlations of the local gamma passing rates with 3%/3 mm, 2%/2 mm, and 1%/2 mm showed moderate correlations to the MLC errors. Similarly to the MapCHECK2 results, the correlations of the non-SABR plans were generally higher than those of the SABR plans.

The *r* values between the gamma passing rates and the gantry angle errors during the VMAT delivery are shown in Table 5 with corresponding *p* values. Weak or no correlations were observed between the gamma passing rates and the gantry angle errors.

**Table 5:** - Correlations between the gamma passing rates and the gantry angle errors

Gamma criteria	MapCHECK2 measurements				ArcCHECK measurements			
	Global gamma passing rates		Local gamma passing rates		Global gamma passing rates		Local gamma passing rates	
	*r*	*p*	*r*	*p*	*r*	*p*	*r*	*p*
TrueBeam STx (SABR + non-SABR)								
2%/2 mm	–	–	–	–	− 0.258	0.010	–	–
1%/2 mm	–	–	–	–	− 0.242	0.015	–	–
TrueBeam STx (SABR)								
2%/2 mm	–	–	–	–	− 0.398	0.002	–	–
1%/2 mm	–	–	–	–	− 0.405	0.001	–	–
1%/1 mm	–	–	–	–	− 0.257	0.048	–	–
TrueBeam STx (non-SABR)								
2%/1 mm	–	–	–	–	0.347	0.028	–	–
1%/1 mm	–	–	–	–	0.333	0.035	–	–

Note: Only *r* values with *p* ≤ 0.05 are shown

The *r* values between the gamma passing rates and the MU delivery errors are shown in Table 6 with corresponding *p* values. For the Trilogy, Weak or no correlations were observed between the gamma passing rates and the MU delivery errors always showing absolute *r* values smaller than 0.34. For the TrueBeam STx, local gamma passing rates of the SABR plans showed weak or moderate correlations to the MU delivery errors (− 0.59 < *r* < − 0.39 with *p* < 0.003) while both the global and local gamma passing rates of the non-SABR plans showed weak or no correlations to the MU delivery errors.

**Table 6:** - Correlations between the gamma passing rates and the MU delivery errors

Gamma criteria	MapCHECK2 measurements				ArcCHECK measurements			
	Global gamma passing rates		Local gamma passing rates		Global gamma passing rates		Local gamma passing rates	
	*r*	*p*	*r*	*p*	*r*	*p*	*r*	*p*
Trilogy (C-series linac)								
3%/3 mm	–	–	− 0.304	< 0.001	–	–	− 0.251	0.003
2%/2 mm	−0.191	0.024	−0.183	0.030	–	–	−0.312	< 0.001
2%/1 mm	−0.238	0.005	–	–	–	–	−0.323	< 0.001
1%/2 mm	−0.211	0.012	–	–	–	–	−0.305	< 0.001
1%/1 mm	−0.216	0.010	–	–	–	–	−0.330	< 0.001
TrueBeam STx (SABR + non-SABR)								
3%/3 mm	–	–	–	–	−0.270	0.007	–	–
2%/2 mm	0.236	0.018	–	–	−0.317	0.001	–	–
2%/1 mm	–	–	–	–	− 0.336	0.001	–	–
1%/2 mm	0.243	0.015	–	–	–	–	–	–
TrueBeam STx (SABR)								
3%/3 mm	–	–	−0.546	< 0.001	–	–	− 0.398	0.002
2%/2 mm	–	–	− 0.456	< 0.001	–	–	− 0.425	0.001
2%/1 mm	−0.452	< 0.001	− 0.581	< 0.001	–	–	− 0.519	< 0.001
1%/2 mm	− 0.491	< 0.001	− 0.441	< 0.001	–	–	− 0.422	0.001
1%/1 mm	− 0.625	< 0.001	− 0.583	< 0.001	–	–	− 0.496	< 0.001
TrueBeam STx (non-SABR)								
1%/2 mm	0.343	0.030	0.323	0.042	–	–	–	–
1%/1 mm	0.369	0.019	–	–	–	–	–	–

Note: Only *r* values with *p* ≤ 0.05 are shown

### Correlations between the gamma passing rates and the MCS_v_

The *r* values between the gamma passing rates and the MCS_v_ are shown in Table 7 with corresponding *p* values. For the Trilogy, both the global and local gamma passing rates showed weak or no correlations except for the local gamma passing rates with 2%/1 mm from the ArcCHECK measurements (*r* = 0.529 with *p* < 0.001). For the TrueBeam STx, no correlations were observed.

**Table 7:** - Correlations between the gamma passing rates and the modulation complexity score

Gamma criteria	MapCHECK2 measurements				ArcCHECK measurements			
	Global gamma passing rates		Local gamma passing rates		Global gamma passing rates		Local gamma passing rates	
	*r*	*p*	*r*	*p*	*r*	*p*	*r*	*p*
Trilogy (C-series linac)								
3%/3 mm	0.193	0.023	0.448	< 0.001	–	–	0.340	< 0.001
2%/2 mm	0.268	0.001	0.392	< 0.001	–	–	0.378	< 0.001
2%/1 mm	0.433	< 0.001	0.352	< 0.001	–	–	0.529	< 0.001
1%/2 mm	0.311	< 0.001	0.238	0.005	–	–	0.282	0.001
1%/1 mm	0.482	< 0.001	0.268	0.001	–	–	0.453	< 0.001
TrueBeam STx								
3%/3 mm	–	–	–	–	−0.243	0.015	–	–
2%/1 mm	–	–	–	–	− 0.196	0.050	–	–

Note: Only *r* values with *p* ≤ 0.05 are shown

### Correlations of the gamma passing rates to the differences in the dose-volumetric parameters between the original plans and the plans reconstructed with log files

The *r* values of the gamma passing rates to the differences in the D_95%_ of the PTVs between the original plans and the reconstructed plans with log files are shown in Table 8 with corresponding *p* values. For both the Trilogy and TrueBeam STx, weak or no correlations were observed between the gamma passing rates and the changes in the D_95%_.

**Table 8:** - Correlations between the gamma passing rates and the differences in the D_95%_ of PTVs between the original plans and the reconstructed plans with log files

Gamma criteria	MapCHECK2 measurements				ArcCHECK measurements			
	Global gamma passing rates		Local gamma passing rates		Global gamma passing rates		Local gamma passing rates	
	*r*	*p*	*r*	*p*	*r*	*p*	*r*	*p*
TrueBeam STx (SABR + non-SABR)								
3%/3 mm	–	–	–	–	–	–	− 0.225	0.025
2%/2 mm	–	–	− 0.264	0.008	–	–	− 0.240	0.016
2%/1 mm	–	–	− 0.238	0.017	–	–	− 0.274	0.006
1%/2 mm	−0.283	0.004	−0.294	0.003	–	–	−0.279	0.005
1%/1 mm	−0.333	0.001	−0.313	0.001	–	–	−0.318	0.001
TrueBeam STx (non-SABR)								
2%/2 mm	–	–	−0.326	0.040	–	–	–	–
2%/1 mm	–	–	−0.340	0.032	–	–	–	–
1%/2 mm	–	–	−0.323	0.042	–	–	–	–
1%/1 mm	−0.421	0.007	−0.436	0.005	–	–	–	–

Note: Only *r* values with *p* ≤ 0.05 are shown

The *r* values of the gamma passing rates to the differences in the V_50%_ of the body structures between the original plans and the reconstructed plans with log files are shown in Table 9 with corresponding *p* values. Same as the results of the PTV D_95%_, weak or no correlations were observed for every case.

**Table 9:** - Correlations between the gamma passing rates and the differences in the V_50%_ of patient body between the original plans and the reconstructed plans with log files

Gamma criteria	MapCHECK2 measurements				ArcCHECK measurements			
	Global gamma passing rates		Local gamma passing rates		Global gamma passing rates		Local gamma passing rates	
	*r*	*p*	*r*	*p*	*r*	*p*	*r*	*p*
Trilogy (C-series linac)								
2%/1 mm	–	–	− 0.166	0.050	–	–	–	–
1%/1 mm	–	–	−0.187	0.027	−0.185	0.029	–	–
TrueBeam STx (SABR + non-SABR)								
3%/3 mm	–	–	–	–	–	–	−0.293	0.003
2%/2 mm	–	–	–	–	–	–	−0.327	0.001
2%/1 mm	−0.253	0.011	–	–	–	–	−0.328	0.001
1%/2 mm	–	–	–	–	–	–	−0.332	0.001
1%/1 mm	−0.254	0.011	–	–	–	–	−0.326	0.001
TrueBeam STx (SABR)								
2%/1 mm	–	–	–	–	–	–	−0.293	0.023
1%/2 mm	–	–	0.264	0.042	–	–	–	–
TrueBeam STx (non-SABR)								
3%/3 mm	–	–	–	–	–	–	−0.351	0.027
2%/2 mm	–	–	–	–	–	–	−0.351	0.027
1%/2 mm	–	–	–	–	–	–	−0.335	0.035

Note: Only *r* values with *p* ≤ 0.05 are shown

## Discussion

In this study, the global gamma passing rates from the MapCHECK2 measurements contradict with those from ArcCHECK for both measurements with Trilogy and TrueBeam STx as shown in Fig. 1. Therefore, no correlations were observed between the global gamma passing rates of the MapCHECK2 and ArcCHECK measurements as shown in Table 3. In addition, except for the global gamma passing rate with 1%/2 mm, the global gamma passing rates with the Trilogy system indicated that the plan delivery accuracy of the prostate VMAT plans were the lowest showing the lowest gamma passing rates although the mechanical errors during the delivery were the lowest as shown in Fig. 3. The MLC positional errors, which are known to be the most dominant mechanical errors affecting the VMAT delivery accuracy, were always the highest for the H&N VMAT plans for both the Trilogy and TrueBeam STx systems [10, 26]. Furthermore, it is known that the modulation degree of prostate VMAT plans is lower than that of H&N VMAT plans with the SIB technique from various previous studies [5, 6, 12, 27]. In the case of H&N VMAT plans, owing to adjacent multiple target volumes with multiple prescription doses, which requires a steep dose gradient between the target volumes, an overlap between the target volumes and OARs, such as parotid glands, and concave-shaped target volumes in the axial view to reduce the doses to the spinal cord and brain stem, the modulation degree of H&N VMAT plans is generally much higher than that of prostate VMAT plans. The values of MCS_v_ in this study also indicated that the modulation degrees of the H&N VMAT plans were the highest while those of the prostate VMAT plans were the lowest since the values of the MCS_v_ decrease as increasing the modulation degree of VMAT plans (Fig. 4) [9]. With the TrueBeam STx system, the global gamma passing rates from the ArcCHECK measurements indicated that the modulation degree of the lung SABR VMAT plans were the highest. In the case of lung SABR VMAT plans, because of a small target volume, a generally round-shaped target volume, and OARs generally located far from the target volume, the modulation degree of lung SABR VMAT is known to be low [28]. This can also be clearly identified in Fig. 3, which shows the lowest MLC positional errors and gantry angle errors. In addition, the MCS_v_ of the lung SABR VMAT plans indicated that the modulation degrees of the lung SABR VMAT plans were the lowest (Fig. 4). Since every VMAT plan analyzed in this study was clinically acceptable according to the previous study showing global gamma passing rates with 2%/2 mm of more than 95% [12], and the power of discrimination of the global gamma evaluation was relatively weak compared to the local gamma evaluation, it seems that the values of the global gamma passing rates could not always show the plan delivery accuracy minutely according to the modulation degree. The contradictory results between MapCHECK2 and ArcCHECK might be attributed to device setup errors or dosimeter response errors. On the contrary, the local gamma passing rates with MapCHECK2 generally showed similar results to those with ArcCHECK, both showing the lowest gamma passing rates of the H&N VMAT plans in general. The local gamma passing rates seem to be more reliable than the global gamma passing rates. The superior reliability of the local gamma passing rates to the global gamma passing rates can also be identified with the higher correlations of the local gamma passing rates between the MapCHECK2 and ArcCHECK dosimeters than those of the global gamma passing rates as shown in Table 3. No correlations were observed between the two dosimeters for the global gamma passing rates (all with *p* > 0.05); however, weak or moderate correlations were observed for the local gamma passing rates with statistical significance (all with *p* < 0.05). Therefore, when using the gamma index analysis to verify the VMAT delivery accuracy, the local gamma index analysis seems more appropriate than the global gamma analysis in general. However, the local gamma index analysis did not always show consistency between the dosimeters and did not always show superior correlations to the mechanical errors (Tables 4, 5, and 6).

To compare the correlations between the global and local gamma passing rates from the MapCHECK2 measurements to those from the ArcCHECK measurements, the tendencies of those two dosimeters were different from each other. Weak or no correlations were observed for the ArcCHECK measurements, while strong correlations were observed for the MapCHECK2 measurements with tight gamma criteria (*r* > 0.7 with *p* < 0.001 with 1%/2 mm and 1%/1 mm). In addition, the correlations of the gamma passing rates from the MapCHECK2 measurements with the MLC positional errors were different from those from the ArcCHECK measurements. For the MapCHECK2 measurements, both the global and local gamma passing rates with 1%/2 mm and 1%/1 mm of the VMAT plans using TrueBeam STx showed moderate correlations with the MLC errors, while the local gamma passing rates with 1%/2 mm showed moderate correlations with the MLC errors for the ArcCHECK measurements.

Since TrueBeam STx delivers VMAT plans more accurately by the integrated control system, i.e., supervisor, than the C-series linac, the mechanical errors during the VMAT delivery of TrueBeam STx were smaller than those of Trilogy. This can be identified in the MLC and gantry angle errors from the log files shown in Fig. 3. Although the MU delivery errors of the TrueBeam STx system were higher than those of the Trilogy system, this was observed only in the SABR VMAT plans, which used a huge amount of MUs. For the fractionated VMAT plans, the MU delivery errors of TrueBeam STx were smaller than those of Trilogy. In addition, both the global and local gamma passing rates of the H&N VMAT plans with the TrueBeam STx system were always higher than those with Trilogy regardless of the gamma criteria.

To review the correlations of the gamma passing rates to the mechanical errors of SABR and non-SABR VMAT plans, the local gamma passing rates of the SABR VMAT plans showed higher correlations to the MU delivery errors than did the non-SABR VMAT plans (absolute values of *r* > 0.39 with *p* < 0.003) while those of the non-SABR VMAT plans showed higher correlations to the MLC errors than did the SABR VMAT plans (absolute values of *r* > 0.36 with *p* < 0.03 except the MapCHECK2 gamma passing rates with 3%/3 mm and the ArcCHECK gamma passing rates with 2%/1 mm). Since the SABR plans delivered relatively larger MUs at each control point than did the non-SABR plans, the MLC speeds during beam delivery were relatively lower than those of the non-SABR VMAT plans (data are not shown). On the contrary, the non-SABR plans delivered relatively smaller MUs at each control point than did the SABR plans, the MLC speeds during beam delivery were relatively higher than those of the SABR plans (data are not shown). Therefore, uncertainty in the MU delivery increased for the SABR plans while uncertainty in the MLC movements increased for the non-SABR plans (Fig. 3). In this respect, we acquired higher correlations of the gamma passing rates to the MU delivery errors for the SABR plans while higher correlations were observed between the gamma passing rates and the MLC errors for the non-SABR plans (Tables 4 and 6).

To review the correlations of the gamma passing rates to the dose-volumetric parameter differences (D_95%_ of the PTV and V_50%_ of the body) between the original treatment plan and the plans reconstructed with the log files, no distinctive correlations were observed same as the results of the previous study [18].

The limitation of the present study is that clinically unacceptable VMAT plans owing to huge discrepancies between the plan and the delivery were not included in the VMAT plans analyzed in this study. Therefore, we could not investigate the tolerance levels for the gamma index analyses in this study. Nevertheless, we found that the gamma passing rates depend on not only the modulation degree or errors in the VMAT plans, but also the types of gamma index analyses, dosimeters, gamma criteria, and types of linacs.

## Conclusions

In this study, correlations between the global and local gamma passing rates varied according to the dosimeter type, linac type, and gamma criteria. In addition, the correlations between the gamma passing rates from different dosimeters were not strong and varied according to the type of the gamma index analysis, linac type, and gamma criteria. The correlations of the gamma passing rates with the MLC errors also varied according to the type of gamma analysis, type of dosimeters, and gamma criteria. Therefore, to utilize the gamma index analysis for the verification of the VMAT delivery accuracy, each institution should carefully establish their own gamma analysis protocol by determining the type of the gamma index analysis and gamma criterion with their own linac and dosimeter.

## Abbreviations

AAA: Anisotropic analytic algorithm
DD: Dose difference
H&N: Head and neck
HD: High-definition
IMRT: Intensity-modulated radiation therapy
IRB: Institutional review board
MCS_v_: Modulation complexity score for volumetric modulated arc therapy
MLC: Multileaf collimator
MU: Monitor unit
OAR: Organ at risk
PRO: Progressive resolution optimizer
QA: Quality assurance
SABR: Stereotactic ablative radiotherapy
VMAT: Volumetric modulated arc therapy
